# Supplementary material for: A UMLS-based spell checker for natural language processing in vaccine safety
Source: BMC Med Inform Decis Mak. 2007 Feb 12;7:3. doi: 10.1186/1472-6947-7-3 (PMC1805499; doi:10.1186/1472-6947-7-3)
Supplement: Additional file 13 — RAP application directory. Sets up directory for RAP (RDF application for PHP) source code [file 1472-6947-7-3-S13.gz › rap/doc/phpdoc/__filesource/fsource_dataset__datasetIteratorAllGraphsMem.php.html]

xml version="1.0" encoding="iso-8859-1"?


File Source for IteratorAllGraphsMem.php


# Source for file IteratorAllGraphsMem.php

Documentation is available at IteratorAllGraphsMem.php

```
1. <?php
2. /**
3. * ----------------------------------------------------------------------------------
4. * Class: IteratorAllGraphsMem
5. * ----------------------------------------------------------------------------------
6. *
7. * @package     dataset
8. */


11. /**
12. * Implementation of a Graph iterator.
13. *
14. * This Iterator should be used in a for-loop like:
15. * for($iterator = $dataset->listGraphs(); $iterator->valid(); $iterator->next())
16. * {
17. *    $currentResource=$it->current();
18. * };
19. *
20. * <BR><BR>History:
21. * <LI>05-03-2005                : First version of this class.</LI>
22. *
23. * @version  V0.9.1
24. * @author Daniel Westphal <mail at d-westphal dot de>
25. *
26. *
27. * @package     dataset
28. * @access    public
29. ***/
30. class IteratorAllGraphsMem
31. {
32. /**
33. * Holds a reference to the associated RDF dataset
34. * @var        object dataset
35. * @access    private
36. */
37. var $associatedGraphSet;

39. /**
40. * The current position
41. * @var        integer
42. * @access    private
43. */
44. var $key;

46. /**
47. * If the current resource is valid
48. * @var        boolean
49. * @access    private
50. */
51. var $valid;

53. /**
54. * The current NamedGraph
55. * @var obejct NamedGraph
56. * @access    private
57. */
58. var $current;


61. /**
62. * Constructor.
63. *
64. *
65. * @param dataset
66. * @access    public
67. */
68. function IteratorAllGraphsMem(&$namedGraphSet)
69. {
70. $this->associatedGraphSet=&$namedGraphSet;
71. $this->rewind();
72. }

74. /**
75. * Resets iterator list to start
76. *
77. * @access public
78. */
79. function rewind()
80. {
81. $this->key = -1;
82. $this->next();
83. }

85. /**
86. * Says if there are additional items left in the list
87. *
88. * @return    boolean
89. * @access    public
90. */
91. function valid()
92. {
93. return $this->valid;
94. }

96. /**
97. * Moves Iterator to the next item in the list
98. *
99. * @access    public
100. */
101. function next()
102. {
103. $this->current = &$this->associatedGraphSet->getGraphWithOffset(++$this->key);
104. $this->valid=($this->current!=NULL);
105. }

107. /**
108. * Returns the current item
109. *
110. * @return    mixed
111. * @access    public
112. */
113. function &current()
114. {
115. return $this->current;
116. }

118. /**
119. * Returns the key of the current item
120. *
121. * @return    integer
122. * @access    public
123. */
124. function key()
125. {
126. return $this->key;
127. }
128. }
129. ?>
```

Documentation generated on Thu, 7 Jul 2005 13:41:46 +0200 by phpDocumentor 1.3.0RC3
